# Medical Gaslighting in Gastroenterology: A Narrative Review From Pediatric and Adult Practice

**DOI:** 10.1016/j.gastha.2026.100961

**Published:** 2026-04-09

**Authors:** Naveed Mustafa, Jahromi Nima, Mönkemüller Klaus, Slonim Anthony

**Affiliations:** 1 Department of Internal Medicine, Virginia Tech Carilion School of Medicine (VTCSOM), Roanoke, Virginia; 2 Department of Pediatrics, Virginia Tech Carilion School of Medicine (VTCSOM), Roanoke, Virginia; 3 Division of Gastroenterology, Department of Internal Medicine, Virginia Tech Carilion School of Medicine (VTCSOM), Roanoke, Virginia; 4 Department of Internal Medicine and Pediatrics, Virginia Tech Carilion School of Medicine (VTCSOM), Roanoke, Virginia

**Keywords:** Medical Gaslighting, Gastroenterology, Patient-Provider Communication, Functional Gastrointestinal Disorders, Diagnostic Delay, Pediatric Care

## Abstract

Medical gaslighting, defined as the dismissal or minimization of patient-reported symptoms in the absence of adequate justification, has emerged as a relevant yet insufficiently examined phenomenon in gastroenterology. Patients with gastrointestinal disorders are particularly vulnerable due to the prevalence of conditions that rely on subjective symptom reporting, evolving diagnostic criteria, and limited availability of definitive biomarkers, especially among functional gastrointestinal disorders and disorders of gut–brain interaction as defined by the Rome IV criteria. This narrative review provides an overview of medical gaslighting in both pediatric and adult gastroenterology, including its conceptual foundations, clinical manifestations, and consequences. Drawing on established theoretical frameworks and contemporary clinical literature, we examine how diagnostic uncertainty, implicit bias, time constraints, and communication gaps contribute to diagnostic invalidation across care settings. Attention is given to populations at increased risk, including children, women, and patients from marginalized racial or socioeconomic backgrounds, as well as to pediatric-specific challenges related to caregiver-mediated symptom reporting and developmental variability. Importantly, this review distinguishes medical gaslighting from appropriate diagnostic uncertainty and from persistent patient concern despite appropriate evaluation, emphasizing the role of transparent diagnostic reasoning and therapeutic alliance. We also highlight validated diagnostic criteria and symptom assessment tools for both functional and inflammatory gastrointestinal disorders as mechanisms to support diagnostic confidence and patient validation. Finally, this review outlines practical, clinically actionable strategies to reduce medical gaslighting in gastroenterology, including bias-aware communication, structured reassurance, use of standardized diagnostic frameworks, and multidisciplinary care models. Addressing medical gaslighting is essential to improving diagnostic accuracy, patient trust, and equitable, patient-centered gastrointestinal care.

## Introduction

### Definition of Medical Gaslighting

Medical gaslighting is defined as a form of psychological invalidation in healthcare in which a patient perceives their symptoms to be dismissed, minimized, or misinterpreted by a healthcare provider, leading the patient to question the validity of their own perceptions and bodily experiences. Medical gaslighting refers to a process in which a person, group, or institution unjustifiably imposes its interpretation of symptoms or illness onto another individual, thereby undermining that patient’s authority to judge their own lived experience. This dynamic destabilizes the patient, leading them to doubt the legitimacy of their symptoms or perceptions and to defer to the clinician or healthcare system as the primary authority. Although the term “gaslighting” originated in the context of intimate partner abuse, it has increasingly been adopted in healthcare settings as patients describe analogous experiences of symptom invalidation.^1^^,^^2^

Medical gaslighting is distinct from diagnostic uncertainty, empiric management, or unavoidable clinical error. Diagnostic uncertainty is inherent to medical practice and remains appropriate when accompanied by transparent reasoning, symptom validation, and a clear plan for reassessment. Poor communication, cognitive errors, implicit bias, or structural constraints such as time pressure may contribute to suboptimal care but constitute medical gaslighting only when they result in persistent dismissal or delegitimization of patient-reported symptoms without adequate explanation or follow-up. For example, attributing recurrent abdominal pain to stress without articulating diagnostic reasoning or escalation criteria differs fundamentally from acknowledging uncertainty while validating patient distress and outlining next steps. In pediatrics, this distinction is particularly relevant when caregiver concerns are minimized as anxiety or inexperience rather than incorporated into ongoing diagnostic reasoning. Women, children, older adults, transgender individuals, patients of color, and those with complex illness are most affected.^3^^,^^4^

By defining medical gaslighting as a form of unjustified epistemic invalidation rather than as a synonym for diagnostic difficulty, this framework preserves conceptual precision and avoids misuse of the term across pediatric and adult gastroenterology.

This narrative review examines how medical gaslighting manifests in pediatric and adult gastroenterology and proposes strategies to recognize and address diagnostic invalidation.

### Narrative Review Methods

A narrative literature review was conducted using PubMed and Google Scholar to identify peer-reviewed articles relevant to medical gaslighting and diagnostic invalidation in gastroenterology. Searches were performed using combinations of the following terms: “medical gaslighting” AND healthcare; “diagnostic invalidation” AND medicine; “functional gastrointestinal disorders” AND stigma; “disorders of gut–brain interaction” AND diagnosis; “Rome IV” AND functional gastrointestinal disorders; “symptom-based diagnosis” AND gastroenterology; “IBS” AND patient experience AND dismissal; “reassurance resistance” AND irritable bowel syndrome; “physician reassurance” AND gastrointestinal disorders; “pain assessment” AND gastroenterology; “chronic abdominal pain” AND stigma; “implicit bias” AND gastroenterology; “gender bias” AND abdominal pain; “racial bias” AND pain assessment; “pediatric diagnostic delay” AND gastroenterology; “functional abdominal pain” AND pediatric diagnosis; “caregiver report” AND pediatric gastroenterology; and “trauma-informed care” AND chronic illness.

Articles were selected based on relevance to pediatric and adult gastroenterology. Given the narrative nature of this review, no formal quality appraisal or systematic screening process was undertaken. The goal was to synthesize conceptual models, illustrative clinical cases, and clinically actionable themes rather than provide an exhaustive or quantitative evidence synthesis.

### Relevance to Gastroenterology

Patients presenting with gastrointestinal (GI) symptoms are uniquely vulnerable to medical gaslighting due to the subjective, episodic, and often nonvisible nature of their complaints. Core GI symptoms, including abdominal pain, bloating, nausea, fatigue, and altered bowel habits, frequently lack definitive biomarkers early in their clinical course, creating diagnostic ambiguity. In adult gastroenterology, this uncertainty is especially pronounced in conditions such as irritable bowel syndrome (IBS), functional dyspepsia, Crohn’s disease, and ulcerative colitis, where nonspecific or fluctuating symptoms may precede confirmatory diagnostic findings. In this context, symptoms may be unintentionally minimized or misattributed to psychological factors such as stress or anxiety, often as an attempt at reassurance. When diagnostic reasoning and follow-up plans are not clearly communicated, these interactions can delay appropriate evaluation and contribute to patient frustration and disengagement from care.^1^^,^^2^

While medical gaslighting in adult and pediatric gastroenterology is driven by shared challenges such as diagnostic uncertainty, subjective symptom reporting, and limited early biomarkers, its expression differs between pediatric and adult care settings. In pediatric gastroenterology, symptom interpretation is often mediated through caregivers and developmental context, shaping how clinical credibility is assigned and how concerns are escalated. In adult gastroenterology, interactions more frequently center on patient self-report, autonomy, and sociocultural expectations, influencing whether symptoms are minimized, psychologized, or prematurely attributed to benign etiologies. The following sections explore these distinctions in greater detail (Table 1).

**Table 1:** Diagnostic Criteria and Validated Assessment Tools for Pediatric and Adult Gastrointestinal Disorders

Disorder	Diagnostic criteria	Symptom severity tools	Quality of life tools
Pediatrics			
Irritable bowel syndrome (IBS)	Rome IV pediatric criteria	Abdominal pain index (API), numeric rating scale (older children)^5^	Functional disability inventory (FDI)^6^^;^ PedsQL GI Module^7^
Functional dyspepsia	Rome IV pediatric criteria	Symptom diaries, API,^5^ Faces Pain Scale (FPS)^8^	FDI,^6^ PedsQL GI Module^7^
Abdominal migraine	Rome IV pediatric criteria	Pain diaries, API,^5^ FPS^8^	FDI,^6^ PedsQL GI Module^7^
Functional abdominal pain–NOS	Rome IV pediatric criteria	API, FPS, pain diaries	FDI,^6^ PedsQL GI Module^7^
Inflammatory bowel disease (IBD)	Endoscopy with histology, imaging, laboratory evaluation	Pediatric Crohn’s Disease Activity Index (PCDAI),^9^ Pediatric Ulcerative Colitis Activity Index (PUCAI)^11^	IMPACT-III quality of life Questionnaire^10^
Adults			
Irritable bowel syndrome (IBS)	Rome IV adult criteria	IBS symptom severity score (IBS-SSS)^12^	IBS-QoL,^13^ SF-36^14^
Functional dyspepsia	Rome IV adult criteria	Patient Assessment of Upper GI Symptoms (PAGI-SYM)^15^	PAGI-QoL^16^
Intestinal migraine	Rome IV adult criteria	Symptom diaries, pain scales	SF-36^14^
Inflammatory bowel disease (IBD)	Endoscopy with histology, imaging, laboratory evaluation	Crohn’s disease patient-reported outcomes signs and symptoms,^17^ Partial Mayo Score (UC)^18^	Inflammatory bowel disease questionnaire (IBDQ),^19^ SF-36^20^

This summarizes commonly encountered functional and inflammatory gastrointestinal disorders across pediatric and adult populations, along with their corresponding primary diagnostic criteria and validated instruments used to assess symptom severity, disease activity, and functional impact or quality of life.

IBS-QoL, IBS Quality of Life; NOS, not otherwise specified; PAGI-QoL, Patient Assessment of Upper Gastrointestinal Disorders Quality of Life; PedsQL, Pediatric Quality of Life Inventory; SF-36, Short Form-36 Health Survey.

### Theoretical Underpinnings

The dynamics of medical gaslighting in gastroenterology can be understood through frameworks examining power and credibility in the clinical encounter. Michel Foucault’s concept of the *medical gaze* describes how medical authority encourages clinicians to interpret patients through a biomedical lens focused on physiology and pathology, often minimizing the patient’s lived experience.^21^ While this enables diagnostic rigor, it may reduce patients to clinical objects rather than participants in their care. In gastroenterology, especially with conditions like IBS or functional pain lacking biomarkers, the medical gaze may predispose clinicians to discount subjective symptoms, leaving patients feeling unheard.

Philosopher Miranda Fricker’s theory of *epistemic injustice* further explains how bias affects credibility in medicine.^22^ *Testimonial injustice* occurs when patient reports are undervalued due to implicit bias, often affecting women, children, and minorities with GI complaints.^3^^,^^4^^,^^23^ *Hermeneutical injustice* arises when systemic gaps limit both patients and clinicians from interpreting or articulating illness, which is common in functional GI disorders where diffuse symptoms lack clear frameworks for validation.

Together, these theories show that gaslighting in gastroenterology reflects structural, not individual, causes rooted in how medical knowledge is produced and applied. Recognizing these patterns underscores the need for patient-centered communication, humility, and addressing power asymmetries that shape diagnostic reasoning.

## Psychosocial and Medical Impacts

Medical gaslighting can cause significant psychosocial harm, including anxiety, depression, and loss of trust in the doctor-patient relationship, which may perpetuate the very symptoms for which the patient sought care.^24^ Patients who feel dismissed may delay care, avoid procedures, or seek repeated opinions for validation. These effects are amplified in vulnerable groups, especially parents or caregivers of children with chronic, complex illness, where repeated invalidation heightens stress and burnout.

Clinically, minimizing or misattributing symptoms can delay diagnosis and proper management, sometimes leading to malnutrition, anemia, or death. In gastroenterology, labeling symptoms as “medically unexplained” may distort decision-making, particularly when frameworks like the Rome IV criteria are ignored or misapplied.^25^ The result is inadequate treatment, prolonged suffering, or disease progression. In children, diagnostic delays may lead to chronic pain, missed school, and developmental or psychological regression.^26^^,^^27^

Diagnostic invalidation also drives disengagement from care, poor adherence, and underuse of support services, worsening outcomes. Providers may experience frustration and burnout when symptoms resist explanation. Confronting these psychosocial and medical effects is essential to rebuild trust, improve accuracy, and strengthen therapeutic relationships in both pediatric and adult gastroenterology.

## Gaslighting in Pediatric Gastroenterology

In pediatric gastroenterology, additional complexities further elevate the risk of medical gaslighting. Unlike adult care, symptom reporting is often indirect, relying on caregiver observations, behavioral cues, or descriptions that a child is “not acting like themselves.” Young children may struggle to articulate symptoms, while caregivers may report subtle or nonspecific signs such as poor feeding, irritability, or changes in behavior. In this context, diagnostic ambiguity can lead to minimization of caregiver concerns, particularly in nonverbal children, first-time parents, or those with neurodevelopmental differences. Medical gaslighting in pediatrics therefore frequently operates through invalidation of the caregiver’s interpretive authority, not solely dismissal of the child’s symptoms.^7^, ^8^, ^9^, ^10^, ^11^

Common pediatric complaints such as picky eating, gastroesophageal reflux, constipation, or vague abdominal pain are often attributed to behavioral or developmental factors without adequate evaluation. Among school-aged children and adolescents, inconsistent symptom reporting, limited recall of stooling patterns, and variable dietary histories further complicate assessment. These dynamics increase reliance on clinician judgment in the absence of objective findings and heighten the risk of diagnostic invalidation when symptoms do not conform to expected patterns.^7^, ^8^, ^9^, ^10^, ^11^

### Diagnostic Challenges in Pediatric GI Care

Pediatric functional GI disorders, now classified under the Rome IV framework as disorders of gut–brain interaction, include IBS, functional dyspepsia, abdominal migraine, and functional abdominal pain not otherwise specified.^25^^,^^28^^,^^29^ Rome IV intentionally emphasizes positive, symptom-based diagnosis following appropriate medical evaluation, rather than exhaustive exclusionary testing. However, misinterpretation of this framework in clinical practice can result in either prolonged over-testing or premature psychologization of symptoms, both of which contribute to repeated encounters without diagnostic closure. This diagnostic ambiguity may inadvertently lead providers to minimize or dismiss parental concerns, particularly in the absence of clear alarm features.

Even in tertiary care settings, diagnostic closure is frequently delayed. In a cohort of children referred for chronic abdominal pain who ultimately met Rome IV criteria for functional GI disorders, the mean symptom duration at referral was 2.8 years, and many patients had undergone multiple medication trials before a positive functional diagnosis was established.^30^ This prolonged period of uncertainty increases the likelihood of repeated clinical encounters in which families perceive dismissal or reassurance without a clear diagnostic rationale.

Abdominal migraine represents a distinct pediatric subtype characterized by recurrent, stereotyped episodes of severe abdominal pain separated by symptom-free intervals. Although it accounts for a smaller proportion of pediatric functional abdominal pain disorders, its episodic nature and reliance on caregiver history for diagnosis make it particularly vulnerable to dismissal when physical examinations and testing are normal between episodes. Failure to recognize abdominal migraine as a legitimate diagnosis may result in prolonged uncertainty, delayed treatment, and repeated reassurance without a management plan.^28^

Functional constipation is another common and frequently underrecognized cause of chronic abdominal pain in children. Abdominal pain related to constipation may persist even when bowel movement frequency appears normal, particularly in the presence of stool withholding behaviors or incomplete stooling histories. In the same tertiary care cohort, functional constipation had not been previously identified in 13.9% of children referred for chronic abdominal pain, underscoring how incomplete stool histories and attribution of symptoms to behavioral factors can delay appropriate treatment and prolong suffering.^28^

Across pediatric chronic abdominal pain referrals, diagnostic delay and “therapeutic churn” are common. Children often experience years of symptoms and multiple medication trials before receiving a clear functional diagnosis, after which management frequently shifts toward education, reassurance, and dietary or behavioral interventions. The failure to name a diagnosis and articulate a transparent diagnostic rationale can lead families to feel dismissed or endlessly cycled through care, reinforcing perceptions of medical gaslighting.^30^

Diagnostic delay is not limited to functional disorders. A systematic review of pediatric inflammatory bowel disease (IBD) found that children experience a median diagnostic delay of several months, with reported ranges of 2–10.4 months for IBD overall, 2.0–18.0 months for ulcerative colitis, and 4.0–24.0 months for Crohn’s disease.^31^^,^^32^

In infants and nonverbal children, reliance on caregiver observation is unavoidable. Subtle changes such as reduced appetite, irritability, or deviations from baseline behavior may signal underlying pathology yet are often attributed to developmental variation or psychosocial stress. Diagnostic complexity is further amplified in children with autism or intellectual disability, where baseline behaviors may already be atypical.^33^ When caregiver concerns are repeatedly discounted, parents may begin to doubt their judgment, delay future care-seeking, and disengage from the healthcare system, impairing early identification of evolving disease and contributing to prolonged suffering, impaired growth, and delayed access to necessary treatment.^34^

Psychiatric comorbidity, particularly anxiety, commonly co-occurs with pediatric functional abdominal pain and is clinically actionable. However, premature attribution of symptoms to psychological causes without validation or a clear diagnostic strategy may itself be perceived as gaslighting. Importantly, acknowledging brain–gut interactions differs fundamentally from dismissing symptoms as “psychological” in the absence of reasoning and follow-up planning.^35^

### Illustrative Case Example

A 12-year-old boy presented with 2 weeks of abdominal pain, bloody diarrhea, fever, and weight loss. At a regional hospital, clinicians attributed his symptoms to infectious enterocolitis and possible dietary or anxiety-related factors. Despite persistent severe symptoms reported by the patient and his parents, concerns were minimized, and he was treated with antibiotics and supportive care without improvement.

As symptoms worsened, he was referred to a tertiary center. Laboratory testing showed anemia and elevated inflammatory markers, with negative infectious stool studies. Colonoscopy revealed colonic ulcerations, and histopathology confirmed Crohn’s disease. After corticosteroid therapy was initiated, the patient improved and later achieved relative disease control on maintenance treatment, with one relapse during follow-up.^32^

IBD affects approximately 10 in 100,000 children in North America, with a rising global incidence. This case, adapted from a published pediatric IBD case series, illustrates how symptom misattribution and premature diagnostic closure can result in extended symptoms, weight loss, and delayed access to necessary treatment.^36^^,^^37^

### Impact on Children and Families

Beyond diagnostic delay, medical gaslighting in pediatric GI care can disrupt physical, emotional, and social development. Children with untreated GI symptoms may miss school, sports, and social events due to pain, urgency, or embarrassment. Chronic absenteeism correlates with poorer academics, weaker peer ties, and more behavioral issues.^27^^,^^38^

Without a diagnosis, families may face delays in obtaining school accommodations under Section 504 or Individualized Education Programs. Some children restrict food to avoid symptoms, risking nutritional deficits, growth delay, or disordered eating.^39^

Parents often advocate persistently in medical and school settings while coping with uncertainty, leading to fatigue, financial strain, and burnout. Siblings may also assume caregiving roles, creating secondary stress and shifting family dynamics (Figure).

**Figure:**
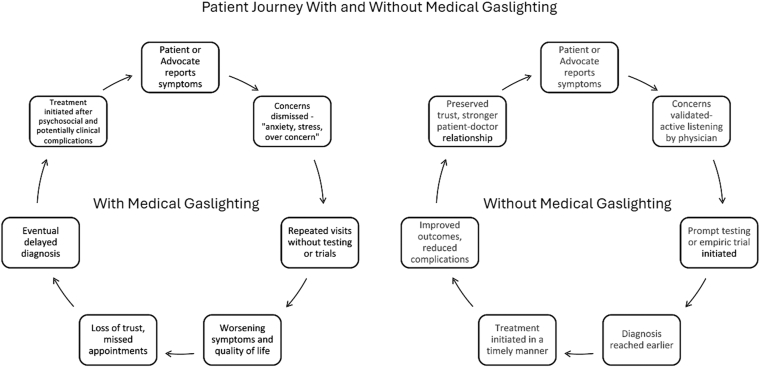
Patient journey with and without medical gaslighting. The dismissed pathway leads to delayed diagnosis and complications, while the validated pathway leads to timely diagnosis, early treatment, and improved outcomes.

## Gaslighting in Adult Gastroenterology

Functional GI disorders, as shown in Table 2, including IBS, functional dyspepsia, and intestinal migraine, are particularly susceptible to diagnostic dismissal due to their reliance on symptom-based criteria and the limited availability of confirmatory biomarkers. Among adults, IBS and functional dyspepsia are the most prevalent functional GI disorders, together accounting for most disorder-of-gut–brain interaction diagnoses encountered in routine gastroenterology practice and frequently co-occurring with other functional symptoms. The high prevalence and overlap of these conditions increase the likelihood of repeated clinical encounters with unrevealing testing, heightening the risk that persistent symptoms are perceived as exaggerated, psychogenic, or nonactionable.^40^

**Table 2:** Gastrointestinal Conditions Commonly Affected by Medical Gaslighting

Condition	Key symptoms	Impact of gaslighting on diagnosis and treatment
Irritable bowel syndrome (IBS)	Abdominal pain, bloating, altered bowel habits	Symptoms often dismissed as anxiety or stress related; minimal workup pursued^1^
Inflammatory bowel disease (IBD)	Chronic diarrhea, weight loss, fatigue	Diagnostic delays due to overlap with functional disorders or assumptions of benign etiology^41^
Celiac disease	Abdominal pain, fatigue, bloating	Frequently misattributed to IBS or dietary sensitivity without confirmatory testing^42^
Functional dyspepsia	Early satiety, epigastric discomfort, nausea	Labeled as psychosomatic or anxiety related; workup often deferred^1^

An emerging literature further highlights the substantial stigma associated with GI conditions, particularly disorder-of-gut–brain interactions such as IBS, gastroparesis, and chronic nausea, which are often viewed as less legitimate than organic disease and more readily attributed to psychological or behavioral factors.^36^ Importantly, diagnostic invalidation is not confined to functional disorders. Adults with organic GI diseases, including IBD,^41^ celiac disease,^42^ microscopic colitis,^43^ mast cell activation syndrome,^44^ and eosinophilic disorders, may also experience delayed or missed diagnoses due to atypical presentations, symptom overlap, or premature diagnostic closure. Similarly, systemic conditions with prominent GI manifestations, such as mastocytosis,^45^ carcinoid syndrome,^46^ multiple autoimmune syndrome,^47^ Cronkhite-Canada syndrome,^48^ and Menetrier’s disease,^49^ are frequently diagnosed years after symptom onset. In adult GI care, time constraints, fragmented follow-up, and the tendency to label patients with persistent, unexplained symptoms as “difficult” may further amplify these dynamics, transforming diagnostic uncertainty into perceived dismissal and reinforcing experiences of medical gaslighting.^1^^,^^50^

### Sociodemographic Influences

Susceptibility to medical gaslighting is influenced by sociodemographic factors, including gender, race, and socioeconomic status. Women and patients from marginalized racial and ethnic groups are disproportionately likely to have their symptoms minimized or attributed to psychological causes, particularly in conditions characterized by subjective pain and limited biomarkers.^3^^,^^4^^,^^23^^,^^51^ Experimental and clinical studies demonstrate that racial bias in pain perception leads clinicians to underestimate symptom severity and provide less thorough diagnostic evaluation and treatment recommendations for Black patients, even when clinical presentations are equivalent.^4^ These credibility gaps are especially salient in gastroenterology, where reliance on patient narrative is central to diagnosis.

Patients with lower socioeconomic status often face barriers to specialty access, limited continuity of care, and challenges related to health literacy, reducing opportunities for longitudinal assessment and effective advocacy. In cases involving overlapping GI and gynecological symptoms such as chronic pelvic or abdominal pain, these biases may result in prolonged diagnostic delay and under investigation, reinforcing perceptions of dismissal.^3^^,^^4^

### Case Narratives

#### Diagnostic delay

A young adult Black woman presented with an 18-month history of postprandial epigastric pain, early satiety, bloating, nausea, and significant unintentional weight loss. As described in a published case report, she sought care across multiple outpatient and emergency settings, where symptoms were repeatedly attributed to anxiety, stress, or IBS following unrevealing routine laboratory testing, negative *Helicobacter pylori* studies, and a nondiagnostic upper endoscopy. Despite progressive functional decline, further evaluation was deferred. Advanced imaging ultimately revealed celiac artery compression consistent with median arcuate ligament syndrome. Diagnostic celiac plexus blockade resulted in immediate symptom relief, and surgical decompression led to resolution of pain and weight restoration.^52^

This case demonstrates how symptom misattribution and bias can contribute to delayed diagnosis of treatable GI disease in adult patients.^4^

#### Reassurance resistance

An adult patient with IBS presented with chronic abdominal pain and ongoing concern for serious underlying disease despite appropriate evaluation and absence of alarm features. The clinician conducted a thorough workup and provided a clear functional diagnosis with education regarding gut–brain mechanisms. However, the patient continued to express worry about alternative diagnoses and symptom-related distress, reflecting limited reassurance despite appropriate care.

This case highlights a key distinction in medical gaslighting: persistent patient concern does not necessarily indicate diagnostic dismissal or clinician error. Even when due diligence is performed, gaps in communication or insufficient therapeutic alliance may limit reassurance. In such scenarios, ongoing anxiety reflects unmet explanatory or relational needs rather than gaslighting, underscoring the importance of sustained trust-building in GI care.^53^

### Impact on Quality of Life

Adults who experience diagnostic invalidation or delayed diagnostic closure may delay or avoid future care, increasing the risk of disease progression, undertreated pain, and functional impairment. Medical gaslighting has been associated with heightened anxiety, depression, and post-traumatic stress-like symptoms, which independently worsen quality of life and clinical outcomes. In GI disease, pain is a major determinant of patient well-being yet is frequently under-recognized or deprioritized in clinical encounters, even in objectively diagnosed conditions.^54^

Persistent symptom dismissal and unmet care needs contribute to chronic pain cycles, reduced participation in work and social activities, and disengagement from the healthcare system. Over time, these effects compound physical morbidity with psychological distress, underscoring medical gaslighting as a mechanism of harm rather than a transient communication failure.

## Recognizing Gaslighting: Key Indicators and Red Flags

### Pediatric Red Flags

In pediatric gastroenterology, early recognition of medical gaslighting is essential to avoid delayed or missed diagnosis. Key indicators as shown in Table 3 below may include multiple visits for unresolved or persistent symptoms, prolonged periods of “watchful waiting” without a clear diagnostic strategy, or hesitancy to pursue appropriate testing despite caregiver concerns. Families may report feeling dismissed, fatigued, or increasingly desperate for answers, signaling strain on the therapeutic relationship.

**Table 3:** Red Flags for Medical Gaslighting in Pediatric Gastroenterology

Red flag	Description	Consequences of gaslighting
Frequent abdominal pain complaints	Recurrent or chronic pain reported without objective findings	Under investigation, mislabeling as psychosomatic
Delayed referrals to specialists	Failure to escalate care despite ongoing or worsening symptoms	Prolonged suffering, delayed diagnosis and treatment
Dismissal of caregiver concerns	Parental observations minimized or attributed to anxiety	Missed diagnoses, caregiver distress, loss of trust

Symptoms in nonverbal or developmentally delayed children are particularly vulnerable to under recognition. Behavioral indicators, such as persistent crying, irritability, or feeding difficulties, may be mischaracterized as developmental variability or parental overreaction. Clinicians who are attentive to these patterns can more effectively validate caregiver input, recalibrate diagnostic approaches, and support timely, appropriate care.^33^

### Adult Red Flags

In adult patients, medical gaslighting may present through repeated misdiagnoses, premature closure, or early attribution of symptoms to psychological factors in the absence of a complete evaluation. Clinical red flags include vague or nonspecific reassurance, time-limited appointments that preclude adequate history taking, and empiric therapies offered without a clear diagnostic rationale.^1^^,^^2^

Patients may report feeling unheard or dismissed, particularly in presentations involving subjective symptoms like abdominal pain, bloating, fatigue, or functional GI complaints. Over time, these patterns may erode the therapeutic alliance and contribute to diagnostic delays, psychological distress, and disengagement from care. Recognizing these dynamics allows for corrective strategies that emphasize validation, transparency, and collaborative diagnostic reasoning.^1^^,^^2^

### Clinical and Ethical Considerations

Gastroenterologists have a professional and ethical responsibility to recognize and address medical gaslighting when they arise. Timely intervention through validation of patient experiences, active listening, and transparent communication can improve diagnostic accuracy, strengthen the relationship, and reduce unnecessary healthcare utilization.

Failure to address these dynamics may perpetuate stigma, contribute to delays in care, and undermine patient trust, ultimately leading to poorer outcomes and increased costs. Incorporating a patient-centered, trauma-informed approach can help mitigate these risks, particularly in the care of individuals with chronic, complex, or poorly understood GI conditions. Trauma-informed care in health care is defined as an approach to care that recognizes the impact of trauma on health and healthcare experiences and adapts clinical practice to promote emotional safety, patient-centered communication, and trust while avoiding retraumatization.^55^

Emphasizing empathy, shared decision-making, and continuity of care is essential for fostering long-term engagement and improving both clinical and psychosocial outcomes.

## Addressing and Reducing Medical Gaslighting in Gastroenterology

### Provider Training and Awareness

Reducing gaslighting begins with clinician education. Provider training should emphasize recognition of the signs of medical gaslighting and its potential psychological and clinical consequences. Awareness of implicit bias, diagnostic uncertainty, and the subtle dynamics of the provider-patient power differential is critical, especially in conditions like IBS, IBD, and functional GI disorders where subjective symptoms predominate.^1^

Educational curricula should discourage labeling patients as “difficult” and promote reflective practice. Validated tools, such as the IBS Quality of Life questionnaire,^56^ IBS Symptom Severity Score,^57^ and the IBD Control Questionnaire,^58^ can help standardize evaluations and guide clinical decision-making. Equally important is avoiding dismissive phrases or minimizing terminology and employing empathetic, patient-centered communication.

Educational curricula should also include lectures on medical gaslighting, conditions that may result in gaslighting, and uncommon medical conditions. One important factor for medical gaslighting is ignorance or lack of knowledge, which can be overcome by education and by exploring potential structural aspects of “functional” diseases. Classic examples of conditions that are now known to be “organic” and not “IBS” are microscopic colitis^43^ and mast cell activation syndrome.^59^

In pediatric gastroenterology, the North American Society for Pediatric Gastroenterology, Hepatology and Nutrition and American Neurogastroenterology and Motility Society (NASPGHAN-ANMS) neurogastroenterology curriculum supports the development of both diagnostic acumen and interpersonal competencies. Core components include differentiating functional from motility disorders, interpreting investigations such as high-resolution manometry, and recognizing psychosocial contributors to GI complaints. Trainees should aim for at least Entrustable Professional Activity Level 3 proficiency, ensuring their ability to deliver accurate, developmentally informed care.^60^

### Improved Diagnostic Strategies

A transparent, patient-centered diagnostic approach lowers the risk of perceived gaslighting. Clinicians should explain the iterative diagnostic process, including data gathering, hypothesis generation, decision-making, and follow-up, to set expectations and build trust when a definitive diagnosis is pending.

Frameworks like the Rome IV criteria^25^ provide reproducible symptom-based pathways for diagnosing functional GI disorders. Instruments like the Patient Assessment of Upper GI Symptom Severity Index^15^ offer additional quantitative assessment tools that can inform monitoring and guide follow-up. Clinicians should strive to balance over-testing and under-testing, maintaining diagnostic rigor without excessive or unnecessary workups. Importantly, patients should be informed about the limitations of current testing, including the reality that the absence of biomarker evidence does not invalidate the legitimacy of their symptoms.

### Patient and Family Empowerment

Engaging patients and families as informed partners in care reduces the risk of gaslighting and promotes shared understanding. This is especially critical in pediatrics, where caregivers often serve as the primary reporters of symptoms for children who are nonverbal or medically complex. Providers should offer high-quality educational resources like the American College of Gastroenterology, NASPGHAN or International Foundation for Gastrointestinal Disorders to support health literacy and informed decision-making.

Encouraging the use of symptom diaries or mobile health tracking tools can help identify symptom patterns, enhance communication, and support diagnostic reasoning. Clinicians should welcome patient questions, allocate ample time for discussion, and support the presence of a caregiver or advocate during visits, strategies that help counter patient isolation and foster a collaborative care environment.^1^

### Collaborative and Interdisciplinary Care

Table 4 shows the ways communication and training can mitigate the effects of gaslighting. Team-based, interdisciplinary models are effective in addressing the multifactorial nature of GI disorders and reducing diagnostic invalidation. Incorporating professionals such as psychologists, dieticians, social workers, and patient advocates facilitates comprehensive care and ensures that both physical and psychosocial dimensions of illness are addressed.

**Table 4:** Strategies for Reducing the Risk of Gaslighting in Gastroenterology

Strategy	Description	Consequences
Empathic communication	Active listening, validating patient concerns	Strengthened trust, improved diagnostic accuracy
Multidisciplinary care teams	Integration of GI, psychology, dietetics, and patient advocacy	Holistic, patient-centered care for complex presentations
Bias awareness training	Education on recognizing and addressing implicit bias	Reduced risk of dismissing symptoms based on demographics

Dietitians contribute critical expertise in nutritional optimization and dietary management, especially for patients with IBS, IBD, or suspected food insensitivities.^61^ Behavioral health specialists support patients in managing stress-related symptom exacerbation, health anxiety, and illness-related functional impairment. Patient advocates assist individuals navigating complex healthcare systems, especially those with lower health literacy or communication barriers. In pediatric settings, school personnel can provide important insights into academic or social disruptions related to GI symptoms and assist in implementing appropriate educational accommodations.^62^

## Conclusion

### Clinical Implications

Medical gaslighting in gastroenterology typically arises not from intentional dismissal but from predictable failures during diagnostic uncertainty, communication breakdown, and misalignment between clinician and patient priorities. Across both functional and organic GI disorders, these failures are modifiable and require deliberate practice-level interventions rather than awareness alone. Clinicians must consistently apply established diagnostic criteria and validated symptom measures, particularly Rome criteria for functional disorders, to support positive, symptom-based diagnoses when biomarkers are absent. Inconsistent use of these tools contributes to prolonged testing or nonspecific reassurance, both of which can invalidate patient concerns. Transparent application of diagnostic frameworks, explicit naming of the diagnosis, clear explanation of reasoning, and defined follow-up expectations reduce uncertainty, legitimize symptoms, and decrease anxiety and unnecessary healthcare utilization.

Second, clinicians must actively counter societal biases rather than unconsciously reproduce them. Studies demonstrate that race and gender influence how pain is perceived, interpreted, and acted upon, with women and racialized patients more likely to have symptoms psychologized or minimized.^3^^,^^4^^,^^23^ In gastroenterology, where symptom reporting is central, implicit bias can shape diagnostic urgency and follow-up decisions. Reducing gaslighting requires intentional reflection on how assumptions about anxiety, coping, credibility, or “difficult patients” may influence care, particularly for patients with chronic pain or nondiagnostic testing.

Third, reassurance must be structured, explanatory, and longitudinal, not declarative. Evidence from IBS populations shows that reassurance alone does not reliably reduce pain, cancer fear, or symptom stressfulness, especially in patients with long symptom duration or comorbid anxiety or depression. Reassurance is most effective when paired with a clear explanation of disease mechanisms, explicit acknowledgment of uncertainty, and a forward plan. Recognizing “reassurance resistance” as a predictable clinical phenomenon, rather than patient noncompliance, helps prevent invalidation even when appropriate workup has been completed.^50^^,^^53^

Finally, clinicians must prioritize what patients prioritize, particularly pain and functional impact. Across GI conditions, pain, not objective disease activity, is the strongest driver of healthcare seeking and quality-of-life impairment. Evidence shows that when clinicians focus narrowly on biomarkers while deprioritizing pain, patients feel unheard even in technically appropriate care. Explicitly acknowledging pain, asking about functional consequences, and engaging in perspective-taking restores alignment and reinforces trust.^63^

### Research and Clinical Priorities

Future research should aim to systematically evaluate the prevalence, drivers, and clinical consequences of medical gaslighting in gastroenterology. Longitudinal studies examining patient outcomes, healthcare utilization, and psychological sequelae will be critical to understanding the broader impact. Additionally, the effectiveness of targeted interventions, such as communication training, validated symptom measurement, and bias reduction strategies, should be rigorously assessed.

Clinically, there is a pressing need to develop and disseminate standardized best practices that integrate patient-centered care into gastroenterology training and continuing medical education. Embedding communication frameworks, shared decision-making models, and interdisciplinary collaboration into routine practice will support more equitable and accurate care.

Ultimately, addressing gaslighting requires a sustained commitment to listening, validating, and advocating for patients, reaffirming the foundational values of trust, empathy, and clinical excellence that underpin the practice of gastroenterology.

## References

[1] Fuss A., Jagielski C.H., Taft T. (2024). We didn't start the fire or did we?—a narrative review of medical gaslighting and introduction to medical invalidation. Transl Gastroenterol Hepatol.

[2] Sood N., Slonim A.D., Society of General Internal Medicine Forum (2025). Leadership loneliness in health care: exploring the causes, consequences, and solutions for a connected future.

[3] Khan K., Tariq N.S., Majeed S. (2024). Psychological impact of medical gaslighting on women: a systematic review. J Prof Appl Psychol.

[4] Boakye P.N., Prendergast N., Bailey A. (2024). Anti-Black medical gaslighting in healthcare: experiences of Black women in Canada. Can J Nurs Res.

[5] Laird K.T., Sherman A.L., Smith C.A. (2015). Validation of the abdominal pain index using a revised scoring method. J Pediatr Psychol.

[6] Kashikar-Zuck S., Flowers S.R., Verkamp E. (2011). Clinical utility and validity of the Functional Disability Inventory among a multicenter sample of youth with chronic pain. Pain.

[7] Varni J.W., Bendo C.B., Denham J. (2014). PedsQL gastrointestinal symptoms module: feasibility, reliability, and validity. J Pediatr Gastroenterol Nutr.

[8] Bieri D., Reeve R.A., Champion G.D. (1990). The Faces Pain Scale for the self-assessment of pain severity in children. Pain.

[9] Hyams J.S., Ferry G.D., Mandel F.S. (1991). Development and validation of a pediatric Crohn's disease activity index. J Pediatr Gastroenterol Nutr.

[10] Cushman G.K., Stolz M.G., Shih S. (2020). Parent IMPACT-III: development and validation of an inflammatory bowel disease–specific health-related quality-of-life measure. J Pediatr Gastroenterol Nutr.

[11] Turner D., Otley A.R., Mack D. (2007). Development, validation, and evaluation of a pediatric ulcerative colitis activity index: a prospective multicenter study. Gastroenterology.

[12] Francis C.Y., Morris J., Whorwell P.J. (1997). The irritable bowel severity scoring system: a simple method of monitoring irritable bowel syndrome and its progress. Aliment Pharmacol Ther.

[13] Abedi A., Kohli P., Montero S. (2023). Content, face, and construct validity of the irritable bowel syndrome quality of life (IBS-QOL) as a measure of bowel-related quality of life in spinal cord injury. Neurourol Urodyn.

[14] Obulapuram R., Narapaka P.K., Esuru C. (2025). Association of gastrointestinal symptoms with health-related quality of life in cancer patients undergoing chemotherapy: a prospective study. Support Care Cancer.

[15] Rentz A., Kahrilas P., Stanghellini V. (2004). Development and psychometric evaluation of the patient assessment of upper gastrointestinal symptom severity index (PAGI-SYM) in patients with upper gastrointestinal disorders. Qual Life Res.

[16] de la Loge C., Trudeau E., Marquis P. (2004). Cross-cultural development and validation of a patient self-administered questionnaire to assess quality of life in upper gastrointestinal disorders: the PAGI-QOL. Qual Life Res.

[17] Higgins P.D.R., Harding G., Leidy N.K. (2018). Development and validation of the Crohn's disease patient-reported outcomes signs and symptoms (CD-PRO/SS) diary. J Patient Rep Outcomes.

[18] Lewis J.D., Chuai S., Nessel L. (2008). Use of the noninvasive components of the Mayo score to assess clinical response in ulcerative colitis. Inflamm Bowel Dis.

[19] Yarlas A., Maher S., Bayliss M. (2020). The inflammatory bowel disease questionnaire in randomized controlled trials of treatment for ulcerative colitis: systematic review and meta-analysis. J Patient Cent Res Rev.

[20] Yarlas A., Rubin D.T., Panés J. (2018). Burden of ulcerative colitis on functioning and well-being: a systematic literature review of the SF-36 health survey. J Crohns Colitis.

[21] Foucault M. (1994).

[22] Fricker M. (2007).

[23] Shane K., Slonim A.D. (2026). Medical gaslighting and its impact on vulnerable populations. J Racial Ethn Health Disparities.

[24] Shapiro D., Hayburn A. (2024). Medical gaslighting as a mechanism for medical trauma: case studies and analysis. Curr Psychol.

[25] Drossman D.A., Hasler W.L. (2016). Rome IV—functional GI disorders: disorders of gut-brain interaction. Gastroenterology.

[26] Woodham A., David A.L., Cooper M. (2021). The experiences of adolescents diagnosed with functional gastrointestinal disorders: an interpretative phenomenological analysis. Clin Child Psychol Psychiatry.

[27] Tersteeg S.M., Borowitz S.M. (2024). School absenteeism as a predictor of functional gastrointestinal disorders in children. Front Pediatr.

[28] Baaleman D.F., Di Lorenzo C., Benninga M.A. (2020). The effects of the Rome IV criteria on pediatric gastrointestinal practice. Curr Gastroenterol Rep.

[29] Thapar N., Benninga M.A., Crowell M.D. (2020). Paediatric functional abdominal pain disorders. Nat Rev Dis Primers.

[30] Martins G.P., Sandy N.S., Alvarenga L.R. (2022). Functional abdominal pain is the main etiology among children referred to tertiary care level for chronic abdominal pain. Arq Gastroenterol.

[31] Ajbar A., Cross E., Matoi S. (2022). Diagnostic delay in pediatric inflammatory bowel disease: a systematic review. Dig Dis Sci.

[32] Mărginean C.O., Meliţ L.E., Mocanu S. (2017). Inflammatory bowel diseases: a burden in pediatrics. Medicine (Baltimore).

[33] Al-Beltagi M., Saeed N.K., Bediwy A.S. (2023). Role of gastrointestinal health in managing children with autism spectrum disorder. World J Clin Pediatr.

[34] Varni J.W., Lane M.M., Burwinkle T.M. (2006). Health-related quality of life in pediatric patients with irritable bowel syndrome: a comparative analysis. J Dev Behav Pediatr.

[35] Cunningham N.R., Kalomiris A., Peugh J. (2021). Cognitive behavior therapy tailored to anxiety symptoms improves pediatric functional abdominal pain outcomes: a randomized clinical trial. J Pediatr.

[36] Taft T.H., Keefer L., Leonhard C. (2009). Impact of perceived stigma on inflammatory bowel disease patient outcomes. Inflamm Bowel Dis.

[37] Benchimol E.I., Bernstein C.N., Bitton A. (2017). Trends in epidemiology of pediatric inflammatory bowel disease in Canada: distributed network analysis of multiple population-based provincial health administrative databases. Am J Gastroenterol.

[38] Logan K., Cuff S., AAP Council on Sports Medicine and Fitness (2019). Organized sports for children, preadolescents, and adolescents. Pediatrics.

[39] Romano C., Dipasquale V. (2021). Nutrition in pediatric gastroenterology. Nutrients.

[40] Abid S., Rehman H., Awan S. (2022). Epidemiology of functional gastrointestinal disorders using Rome III adult questionnaire: a population-based cross-sectional study in Karachi, Pakistan. PLoS One.

[41] Vavricka S.R., Spigaglia S.M., Rogler G., Swiss IBD Cohort Study Group (2012). Systematic evaluation of risk factors for diagnostic delay in inflammatory bowel disease. Inflamm Bowel Dis.

[42] Fuchs V., Kurppa K., Huhtala H. (2014). Factors associated with long diagnostic delay in celiac disease. Scand J Gastroenterol.

[43] Münch A., Aust D., Bohr J., European Microscopic Colitis Group (2012). Microscopic colitis: current status, present and future challenges—statements of the European Microscopic Colitis Group. J Crohns Colitis.

[44] Jung J., Suman M.C. (2025). The impact of delayed diagnosis on quality of life in patients with mast cell activation syndrome. Cleve Clin J Med.

[45] Sokol H., Georgin-Lavialle S., Canioni D. (2013). Gastrointestinal manifestations in mastocytosis: a study of 83 patients. J Allergy Clin Immunol.

[46] Toth-Fejel S., Pommier R.F. (2004). Relationships among delay of diagnosis, extent of disease, and survival in patients with abdominal carcinoid tumors. Am J Surg.

[47] Cojocaru M., Cojocaru I.M., Silosi I. (2010). Multiple autoimmune syndrome. Maedica (Bucur).

[48] El Hayek M., Zaver S., Huang Y. (2025). Cronkhite–Canada syndrome: an inflammatory bowel disease mimic. Am J Gastroenterol.

[49] Almazar A.E., Penfield J.D., Saito Y.A. (2021). Survival times of patients with Ménétrier’s disease and risk of gastric cancer. Clin Gastroenterol Hepatol.

[50] Šojat D., Volarić M., Keškić T. (2024). Putting functional gastrointestinal disorders within the spectrum of inflammatory disorders can improve classification and diagnostics of these disorders. Biomedicines.

[51] Hoffman K.M., Trawalter S., Axt J.R. (2016). Racial bias in pain assessment and treatment recommendations, and false beliefs about biological differences between black and white patients. Proc Natl Acad Sci U S A.

[52] Ray R., Raval D., Patel K. (2025). When pain is silenced: missed diagnosis of celiac artery compression syndrome in a young Black woman due to racial and gender bias. Am J Gastroenterol.

[53] Schmulson M.J., Ortiz-Garrido O.M., Hinojosa C. (2006). A single session of reassurance can acutely improve the self-perception of impairment in patients with irritable bowel syndrome. J Psychosom Res.

[54] Sundas A., Sampath H., Lamtha S.C. (2024). Psychosocial quality-of-life correlates in functional gastrointestinal disorders. Rev Gastroenterol Mex (Engl Ed).

[55] Roberts S.J., Chandler G.E., Kalmakis K. (2019). A model for trauma-informed primary care. J Am Assoc Nurse Pract.

[56] Andrae D.A., Patrick D.L., Drossman D.A. (2013). Evaluation of the Irritable Bowel Syndrome Quality of Life (IBS-QOL) questionnaire in diarrheal-predominant irritable bowel syndrome patients. Health Qual Life Outcomes.

[57] Roalfe A.K., Roberts L.M., Wilson S. (2008). Evaluation of the Birmingham IBS symptom questionnaire. BMC Gastroenterol.

[58] Bodger K., Ormerod C., Shackcloth D. (2014). Development and validation of a rapid, generic measure of disease control from the patient's perspective: the IBD-control questionnaire. Gut.

[59] Akin C. (2017). Mast cell activation syndromes. J Allergy Clin Immunol.

[60] Khlevner J., Rosen R., Ambartsumyan L. (2021). Development of entrustable professional activities and standards in training in pediatric neurogastroenterology and motility: North American Society for Pediatric Gastroenterology, Hepatology and Nutrition and American Neurogastroenterology and Motility Society Position Paper. J Pediatr Gastroenterol Nutr.

[61] Pessarelli T., Bianchi L., Pugliese D. (2022). The low-FODMAP diet and the gluten-free diet in the management of functional abdominal bloating and distension. Front Nutr.

[62] Sagawa T., Okamura S., Kakizaki S. (2013). Functional gastrointestinal disorders in adolescents and quality of school life. J Gastroenterol Hepatol.

[63] Huisman D., Andrews E., Williams A.C.D.C. (2024). Patients and clinicians have different priorities when discussing pain in the IBD clinic. BMJ Open Gastroenterol.

